# Emotions and the War

**Published:** 1918-08

**Authors:** 


**The Emotions and the War.** Professor DUPRÉ, Member of the Academy of Medicine, Major of first Classe in the Army, said that for several years, he has individualized under the name of " Emotive Constitution " a peculiar mode of lack of poise of the nervous system, characterized by diffuse erethism of the general sensibility, sensorial as well as psychic; by the insufficiency of motor inhibition reflex and voluntary: and by reactions abnormal in their degree, diffusion, and duration, and their disproportion to the causes which provoke them.

Hypersensitivity, normal in the infant, very frequent in the child (infantile neurosity), disappears in the adult by the progressive development of the inhibitory tracts which assure the equilibrium and stability of the nervous system.

Most frequently constitutional morbid emotivity of hereditary origin may be acquired and arise from pathogenetic causes; from infections toxic and traumatic; and from intense and repeated commotions and emotions.

Emotion, indeed, often renders the nervous system sensitive to ulterior conditions and by a sort of emotive anaphylaxis may create constitutional emotivity. In opposition to these cases one may observe a progressive familiarity with a whole series of emotions, thus conferring, by repetition of effective shocks, a remarkable *emotive immunity*.

The emotive constitution is characterized by a double series of permanent signs, physical and psychical.

A. — Physical Signs.

1. Diffuse hyperreflectivity of the tendons, cutis, and the pupils. Sensorial hyperesthesia with quick, extended, and prolonged motor reactions, especially mimic and vocal.

2. Lack of motor equilibrium : spasmodic visceral movements; pharyngoesophagia, gastroenteric spasm, cytispasm with pollakiuria.

Emotive trembling in its many forms, tremor of the extremities, starting, jumping, shivering, chattering of the teeth, stammering, occasional myoclonies, tics, etc.

3. Functional inhibition and transitory motor impotence — giving way of the legs, relaxation of the sphincters.

4. Circulatory Disturbances : occasional tachycardia, often permanent and paroxystic instability of the pupils, alternate peripheral vasoconstriction and vasodilatation. The relation of the circulatory disturbance to certain forms of arterial hypertension has yet to be determined, above all in subjects not suffering from arteriosclerosis or from renal lesions.

5. Loss of normal balance : objective variations, appreciable by the local thermometer and subjective sensations of heat and cold principally in the extremities.

6. Glandular disturbances : variations, spontaneous or caused by the emotional shocks of the sudoram, salivary, lacrymal, gastro-intestinal, urinary, genital and biliary secretions.

7. Disturbances of the intervisceral reflexes, by association of spasms, secretory disturbances; functional excitation or inhibition causing abnormal reflexes of one organ or another, along the vago-sympathetic or cerebrospinal route.

### B. — Psychical Signs.

Impressionability, nervousness, state of uneasiness, anxiety, irritability, impulsiveness — these morbid states, more or less continuous or intermittent, often paroxysmal, alternate and intermingle and constitute a permanent foundation, a soil in which appear and develop the emotive syndromes : timidity, scruples, doubt obsessions, phobia, anxious states, either simple or delirious, anguish, psychosexual emotive anomalies.

For the most serious cases there are fits of anxious melancholy, chronic states of obsessions, passing to incurable or auto-accusation hypochondria, negativism.

Constitutional emotivity, which, moreover, may be allied to normal or superior states of mind and of feeling, is frequently associated with other morbid states. Debility or lack of balance in the intelligence, sentiments, and will; various anomalies of the visceral sensibility, of mobility, or of intuition. Emotive psychoneurosis is clearly distinct from neurasthenia and hysteria, with which, however, it shows interesting points of resemblance as to association and succession.

The speaker could not in so short a time set forth the points of resemblance of morbid emotivity with neurasthenia, understood, as it should always be, in the primitive sense of Bend and Charcot. Neither could he set forth the relation between emotivity and hysteria.

The war, powerful cause of fatigue and emotion, has multiplied the cases of psychoneurosis, of exhaustion, and of emotivity. However frequent these cases may have been relatively, they are very rare, from an absolute point of view, in proportion to the enormous number of combatants. Among the millions of soldiers of the great war, almost all have not only acquired a remarkable immunity but, moreover, by the development of the highest sentiments of patriotism and of duty, these men submitting voluntarily to the most severe discipline have raised themselves to the most passionate exaltation of courage and of contempt of death.

Among soldiers who by a personal predisposition have succumbed to emotive shocks due to the circumstances of war, one has observed cases of morbid cowardice with flight and desertion. When the psychiatrist has established the pathological motive in these crimes the subject ought to be declared immediately irresponsible before a court-martial and should be discharged.

Discharge should also be given in the case where emotive shock has caused in the victim psychic disturbances, serious and lasting mental confusion, post confusional dementia of a catatonic or hebephrenic nature.

